# Regional cancer care leads´ and patient representatives´ perspectives on national governance and organisation of palliative cancer care

**DOI:** 10.1186/s12913-026-14691-z

**Published:** 2026-05-13

**Authors:** Cecilia Larsdotter, Anna O´Sullivan, Stina Nyblom, Anneli Ozanne, Carl Johan Fürst, Joakim Öhlén

**Affiliations:** 1 Department of Nursing Sciences, Sophiahemmet University, P.O Box 5605, Stockholm, SE- 114 86 Sweden; 2 Department of Health Care Sciences, Marie Cederschiöld University, Stockholm, Sweden; 3 Palliative Centre, Sahlgrenska University Hospital, Gothenburg, Västra Götaland Region Sweden; 4 Institute of Health and Care Sciences, Sahlgrenska Academy, University of Gothenburg, Gothenburg, Sweden; 5 Department of Neurology, Sahlgrenska University Hospital, Gothenburg, Sweden; 6 Faculty of Medicine, Lund University, Lund, Sweden; 7 Institute for Palliative Care, Region Skåne, Lund, Sweden; 8 Centre for Person-Centred Care (GPCC), University of Gothenburg, Gothenburg, Sweden

**Keywords:** Early integration, Governance, Interpretive description, Organisation, Palliative cancer care, Palliative medicine, Policy

## Abstract

**Background:**

Ensuring that national governance and healthcare systems include the organization and provision of palliative care in all care settings for all patients in need is a global imperative. In cancer care, early palliative care can improve quality of life for patients and families and receiving specialised palliative care increases opportunities for care and death in the preferred place, which for a majority is the own home. In Sweden, national general health policy only vaguely addresses palliative care, leading to the introduction of specific guidelines in 2013. These coexist with national disease-specific guidelines for cancer care that are ambiguous in their conceptualisation and inclusion of palliative care. From a governance and organisation perspective, place of death serves as a key indicator of palliative care infrastructure and organisation. Since policy initiation in 2013, hospital has remained the predominant place of death in Sweden. Further, regional disparities persist and are influenced by factors such as age, sex, and access to specialised services, pointing to inequities and unsatisfactory governance and organisation of palliative care. The aim of this study was to explore the perspectives of cancer care leads and patient representatives on national governance and organisation of palliative cancer care.

**Methods:**

Interpretive description methodology was used to generate and inductively analyse data from group discussions and individual interviews with 36 cancer- and palliative care leads, and patient representatives from the six Swedish regional cancer centres.

**Results:**

The analysis revealed patterns of interdependent conditions that, from the perspectives of cancer care leads and patient representatives shape the governance and organisation of palliative cancer care and seemingly trigger ambiguity regarding responsibilities and inequalities in service provision: *Multilevel knowledge gaps about palliative care; Challenges and complexities of providing palliative care in a fragmented healthcare system;* and *Policy impact and ownership problems.*

**Conclusions:**

The study revealed significant challenges in national palliative cancer care governance, primarily due to a multilevel knowledge gap about palliative care, a fragmented healthcare system, and non-directive national policies. Integration of mandatory national minimum requirements for palliative care in national policy, and clearer standards for palliative care resource allocation are needed. Comprehensive strategies and coordinated efforts that can be uniformly implemented across regions, and establishing national collaborative spaces for regional stakeholders are essential to ensure equitable and timely access to palliative care for all patients with advanced cancer.

**Supplementary Information:**

The online version contains supplementary material available at 10.1186/s12913-026-14691-z.

## Background

According to the WHO guidelines [1], ensuring that national governance and healthcare systems include the organisation and provision of palliative care in all care settings for all patients in need is a global imperative. One large group of such patients is people dying of cancer. Recent global statistics indicate that approximately 10 million deaths are due to cancer and twenty million new cancer cases arise each year. Europe accounts for about two million of these cases, with lung, colorectal, breast, and pancreatic cancer being the most common [2, 3]. It is predicted that these figures will have increased by 30% in 2050 [2, 3]. Prolonged survival from cancer due to better living conditions and more advanced treatments, including more advanced cancer treatments, has resulted in increasingly ageing cancer populations with high prevalence of long-term illness and palliative care needs. Consequently, the World Health Organisation (WHO) underscores the critical importance of developing both robust cancer prevention strategies [4] and comprehensive palliative care [1], highlighting these as urgent public health priorities. Palliative care should be integrated in national healthcare systems at all levels. It should be provided either as non-specialised (in e.g. hospitals, primary care or nursing homes that can be supported by palliative care consultation teams) and as specialised services (in e.g. hospices, palliative care units, or specialised palliative home care) [1].

Early introduction of palliative care in cancer care refers to a palliative approach aiming towards achieving quality of life in parallel with disease-directed antineoplastic therapies. In this way, palliative care is extended beyond its traditional association with late phase and care for the dying. Incorporating palliative care into a broader spectrum of cancer care and addressing patients’ changing needs have shown the potential to optimise quality of life for patients and their family members throughout the illness trajectory [5–9]. However, barriers to early introduction of palliative care in oncology have been identified. These include inadequate funding, absence of standardised referral guidelines, insufficient infrastructure (particularly in rural areas), misconceptions about palliative care being solely for end-of-life situations, lack of palliative care training for health professionals, poor coordination between oncology and palliative care teams, and the reluctance of oncologists to integrate a palliative approach parallel to curative treatments [10–13].

The inclusion of palliative care in cancer policy has been encouraged internationally [14]. Healthcare policies aim to drive the direction of investment and action to prevent or treat illness and relieve suffering from incurable disease. In Sweden, general health policy only vaguely incorporates palliative care. As a result, specific national guidance [15] and guidelines [16] for palliative care were developed and introduced in 2013. National cancer disease-specific guidelines (colorectal cancer, breast cancer etc.) have been established, and these are being developed by cancer care leads in the six Swedish healthcare regions. However, upon examining these guidelines, we found various conceptualisations of palliative care that are likely to create ambiguity, and in some documents, information about palliative cancer care was sparse [17].

From a public health palliative care perspective, place of death distribution has for the past decades been considered to provide a robust indication of the state (i.e. infrastructure and organisation) of palliative care in a country [18]. Although more recent critiques have questioned its value as a direct measure of care quality [19], death in the preferred place is still used as a quality indicator in many countries, including Sweden [15, 16, 20], as it aligns with core principals of person-centred care. It is well known that individual (diagnosis, age, sex), socioeconomic (marital status, living situation, educational attainment) and geographic (residential area, level of urbanisation) factors, and health care utilisation (number of hospital transfers or emergency visits at the end-of-life, access to specialised palliative services) impact where people die [21–23] .

In European countries, between 18% and 76% of all cancer deaths occur in hospital [24, 25]. Previous research has shown that patients with cancer receiving specialised palliative care are more likely to die at home than patients receiving non-specialised palliative care [26], and that potential futile treatment of people dying from cancer aged 65 and above is associated with a higher proportion of hospital deaths [27]. In Sweden, which is the context for this study, approximately 90 000 people die each year and of those about 25% die with cancer as an underlying cause of death [22]. With an interest in the potential impact of the national palliative care policy documents from 2013 [15, 16] on palliative cancer care, we therefore investigated longitudinal trends in place of death for Swedish citizens dying of cancer from 2013 to 2019 [23]. We found a statistically significant trend for a decreasing number of hospital deaths, except for those residing in nursing homes for which no trend was identified, and hence, hospital remains the most common place to die (48.7%), followed by nursing home (25.6%) and own home (23.5%). Moreover, we found large cross-regional variations associated with cancer type, sex, age, marital status, and utilisation of specialised palliative services, all significantly pointing to inequities and questions about national governance of palliative care in the cancer context across the country.

Globally, national health services vary in their organisation and implementation of palliative care. The New Essentials public health model for palliative care [28] suggests integrating four key components: specialised palliative care, non-specialised palliative care, compassionate communities, and civic end-of-life care that educates and engages the public. These components are present to varying degrees in all national healthcare systems. The challenge for each country is to implement the model according to its national population and organisational needs. The four components are interdependent, and effective palliative care relies on their integration and strong collaboration. Reviewing the existing literature about the governance of palliative care perspective primarily in Europe, challenges related to decentralised governance structures, resource limitations, including funding shortages, lack of standardised training and inconsistent curricula, insufficient integration with existing health and social care systems and poor collaboration between policymakers and healthcare professionals to ensure quality control are presented [29–31].

The international literature has made some assertions about the level of palliative care integration in the Swedish healthcare system. Depending on the methodology used, these vary, with some authors asserting palliative care integration is in its infancy, including for patients with cancer [27], while others state that palliative care is well-integrated in the system [32–34]. With an interest to provide a broader perspective on our findings from the trends in place of death [22]- and document studies [17] pertaining to people dying of cancer, this study seeks to contribute to more solid understanding of mechanisms affecting the national state of palliative cancer care.

## Methods

The aim of this study was to explore the perspectives of cancer care leads and patient representatives on national governance and organisation of palliative cancer care.

The study is the third part of a wider, multiple-methods research project on the state of palliative cancer care in Sweden. The project has previously investigated: (i) trends in place of death within the Swedish total population [23], the cancer population [22]; and (ii) inclusion of palliative care in national disease-specific policy documents for cancer in adults [17].

In this third study (clinical trial number: not applicable), we employed an interpretive description methodology [35] to generate knowledge that informs and translates into practice. Interpretive description is designed to facilitate the generation of knowledge about contextuality and complexities. It acknowledges that data is shaped by the interaction between researchers and participants, and that preunderstandings of the phenomenon under study – governance of palliative cancer care – guide the research process towards generating knowledge about the current state of palliative cancer care.

### Setting: The Swedish healthcare system

A feature of the Swedish healthcare system is its decentralised structure. Under the Ministry of Health and Social Affairs, The National Board of Health and Welfare (NBHW) is responsible for implementing policies and guidelines, supporting healthcare providers to maintain compliance with national standards. The twenty-one regions in the country are responsible for planning, financing, and providing hospital and primary care, while the 290 municipalities have the same responsibility for municipality care, which includes care for older people. The regions are organised into six health care regions to facilitate cooperation in tertiary care, for example, for cancer [36]. A joint organisation, The Swedish Association of Local Authorities and Regions (SALAR), facilitates national and inter-regional collaborations [37] but the decentralised structure provides significant autonomy to regions and municipalities in the planning and organisation of health care. In addition, six Regional Cancer Centres (RCC) support the development of cancer care according to the national cancer strategy and the EU’s cancer plan [38]. These centres, which are responsible for national cancer guidelines, are led by executives and cancer leads (experts on the different cancer types) and include patient representatives. Currently there is an ongoing national healthcare reform that focuses on transforming the healthcare system to be more person-centred and integrated. This reform, known as ‘Good and Close Care,’ aims to make primary care the central hub of the healthcare system. By doing so, it seeks to ensure that care is more accessible, coordinated, and tailored to the needs of each individual patient [36]. The significance of place of death patterns is intricately linked to this strategic health service reform, which aims to shift as much care as possible, including palliative care, into people’s ordinary and nursing homes. It also aligns with preferences regarding place of death in the Swedish population [38].To achieve high-quality, equitable, knowledge-based, and resource-efficient care, the decentralised organisation is linked to a national knowledge governance organisation. This consists of twenty-six national programme areas, which include one for cancer (consisting of the regional cancer centres in collaboration) and one for old people’s health and palliative care. One example of their initiatives is the development of a person-centred and integrated palliative care pathway [39], which reflects early integrated palliative care. Some regions and hospitals have also developed palliative care knowledge centres, aiming to disseminate knowledge promoting palliative care developments. While national quality registers exist for most cancer diseases, these have limited palliative care data [17]. A specific national quality register; however, monitors palliative care quality indicators during the final week of life, such as end-of-life conversations and symptom relief [40].

### Sampling and participants

A purposive sample of 157 potential participants, including 119 cancer leads (i.e. clinicians with cancer type specific expertise), 13 RCC executives or palliative care leads (i.e. experts in palliative care) and twenty-five patient representatives from the six regional cancer care organisations were identified through RCC’s website and national patient association websites. Inclusion criteria were cancer care leads (including authors of and experts on cancer care policy documentation), RCC executives, and patient representatives involved in the cancer care organisation who had experience of and the mandate to drive development and implementation of cancer and palliative care policy. Twelve of the invitees actively declined participation, while two agreed to participate but never did, for reasons unknown.

All the identified eligible cancer care leads were invited via e-mail to participate in an online group discussion. An informed consent form and written information were attached to the invitation, assuring confidentiality and the right to withdraw from the study at any time without explanation. Two reminders were sent out. Those who chose to participate (*n* = 36) returned a signed informed consent form and received an invitation to an online discussion. Participants who were interested in participating but unavailable for the scheduled group discussions were offered a separate individual interview. A few participants from the group discussions and individual interviews were also interviewed twice to follow up on topics that had arisen during the first occasion. All participants were informed that the interview would be recorded, and that all data would be de-identified and presented on group level.

### Data generation

**Fig. 1:**
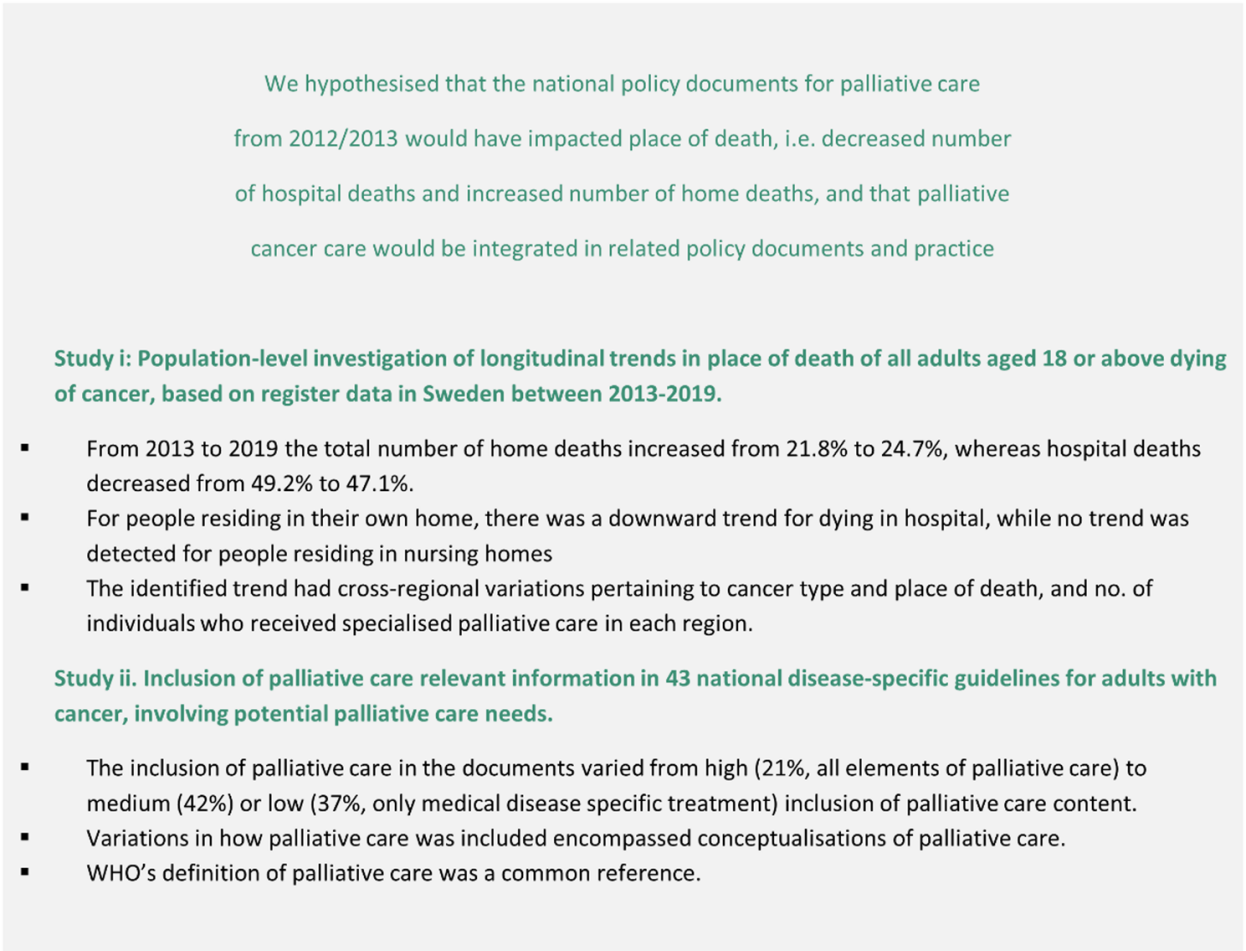
Brief illustration of results from previous studies [17, 22] presented in the introduction to trigger group discussions and individual interviews

Two researchers were present for the group discussions; one as the facilitator and one as an observer, who also posed elaborating questions. One researcher held the individual interviews. The first individual interviews and group discussions started with a brief power point presentation. First, brief comments on the national policy for palliative care was introduced to the participants as a starting point for discussing the current governance and organisation of palliative cancer care in the country, with particular attention to improved quality of life, equity, early integration and. Then results from the cancer specific studies [17, 22] within the wider research project were summarised. The intention was to trigger reflection and facilitate discussion about the state of palliative cancer care in Sweden (Fig. 1).

After the presentation, open discussions were initiated with the following thematic questions (also available as Supplementary): *How do you view these results based on the national healthcare goal of equal care? What can affect the conditions for palliative cancer care nationally*,* and potentially explain regional differences in terms of place of death? What impact does the national knowledge governance of health care for palliative care have? What strategies do you see as successful for governance of palliative care within the region and cancer type you represent?* The discussions in each group evolved around these questions in diverse ways and the participants shared examples, reflections, explanations and asked each other questions. The researchers also asked follow-up questions and invited participants to clarify and elaborate.

### Data analysis

Audio recordings were first transcribed automatically and were then checked and adjusted manually. All data was read to obtain an overall understanding of the discussion on governance and organisation of palliative cancer care in relation to the overall aim of the study. Then, the transcripts were broadly coded by identifying meanings and variations in the contextual descriptions related to governance and organisation of palliative cancer care. The broad codes were analysed for patterns by considering how they were connected to or overlapped each other, and what characterised the data. These patterns were then interpreted through a process of asking questions of the data, such as, *what is the underlying meaning of this*,* what does it mean for the governance of palliative cancer care?* Enhanced understanding was also reached through parallel reading of empirical studies and relevant text related to policy, organisation, and governance of palliative cancer care. The interpretations of data were iteratively discussed and revised within the research group for clarification and further development of the analysis [35].

The Swedish Ethical Review Authority approved to the study (no. 2022-03035-01).

## Results

In total, 30 (23 physicians/7 nurses) cancer care leads, RCC executives or palliative care leads, and six patient representatives participated in the study. All six healthcare regions were represented in at least one of the group discussions. The cancer types represented by the participants were: blood cancer; bone and soft tissue sarcoma; sarcoma; breast cancer; oesophageal-ventricular cancer; gynaecological cancer; liver pancreas and biliary tract; lung cancer; prostate cancer; colon and rectum cancer; and bladder and urinary tract cancer.

In total, sixteen (16) interviews or group discussions were performed; ten were individual interviews and six were group discussions with 3–6 participants. Three of the individual interviews were repeat interviews with key persons who had already participated in a group discussion. The aim of two of these repeat interviews was to generate further understanding of governance and organisation, while the third repeat interview was with a patient representative to gain further understanding from a patient perspective. The length of the interviews ranged from 45 to 60 min.

**Fig. 2:**
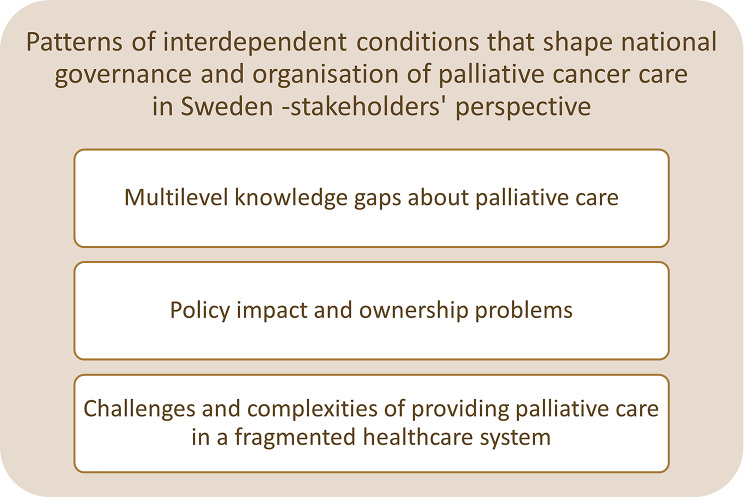
Patterns of interdependent conditions that shape national governance and organisation of palliative cancer care in Sweden from the perspectives of regional cancer care leads and patient representatives

The analysis revealed patterns of interdependent conditions that shape national governance and organisation of palliative cancer care: *Multilevel knowledge gaps about palliative care; Challenges and complexities of providing palliative care in a fragmented healthcare system;* and *Policy impact and ownership problems* (Fig. 2). To protect participants’ integrity, the study omitted to specify their regional belongings and roles within the RCC organisation. Participants’ suggestions for the development of equality in palliative cancer care in Sweden are embedded.

### Multilevel knowledge gap regarding palliative care

In discussing the state of palliative care in the regions, the participants asserted there were misconceptions or even a lack of knowledge about palliative care at political and governance level, as well as at a public level and in healthcare service level. Participants argued that *on a political and governance level*, insufficient understanding of the necessary skills, resources and responsibilities required on different levels in the healthcare system severely impact organisational changes and political decisions. This was expressed as a barrier to effective organisation of non-specialised and specialised palliative care services, and contradicts national palliative care policy, as explained here:

The discrepancy lies with the ones in charge, primarily that’s to say the politicians, but perhaps even more so with these civil servants – they’re the ones who largely don’t understand this… it’s mainly these bureaucrats up at the regional offices who don’t understand how things work. There are huge differences between municipal areas or municipalities within one large regional remit, such big differences in equal treatment and resources, but like I said, that’s nothing to do with the everyday stuff, it’s to do with the politicians and civil servants.

[Group discussion 5, Participant A]

[Participant B responds]

We can devise the nicest-looking care programmes but if no-one reads them or doesn’t accept them as fact because they’re following their own agendas, then it turns into something else. They’re kind of not a controlling factor – a region can choose not to follow a care programme because it’s too expensive. So, it’s just like [refers to participant A] says, it’s the leadership and management and organisation within the region that affects the palliative care.

The participants emphasised the need for decision-making in palliative cancer care to be based on robust research and expertise, rather than being driven by political agendas. To achieve this, they stressed the importance of involving researchers, patient representatives, and knowledge-based government organisations in the political and governance processes, especially when making infrastructural or organisational changes to impact on palliative care.

On a *healthcare service level*, one barrier to the early introduction of palliative care frequently mentioned by participants was the problem of early referrals to specialised palliative care services. The participants said these referrals were often rejected due to patients’ absence of severe symptoms, i.e. not being eligible for specialised palliative care.

The participants acknowledged that lack of comprehensive training in palliative care in the cancer context was a barrier to effective palliative care integration, especially for patients in the initial stages of metastatic cancer. They also emphasised a need for physicians and other professionals in cancer care to have continuous education and training, along with those working in primary care settings and nursing homes, particularly due to the ongoing implementation of the person-centred and integrated care reform. Very few participants reflected on the early introduction of non-specialist palliative care as an actual responsibility for oncologists or other cancer specialists. Instead, participants said that these specialists may often feel a lack of agency and responsibility when it comes to the non-specialised and early introduction of palliative care and that this reluctance is partly due to the belief that discussing palliative care might diminish patients’ hope, leading to hesitancy in initiating end-of-life conversations or referring patients to specialist palliative care.

Finally, on a *public level*, the study participants pointed to a pervasive misconception that palliative care is synonymous with end-of-life care for the dying, which creates fear and resistance among people. Paradoxically, they told, when patients are in a severe state of illness, they are often too weak to advocate for palliative care themselves. Hence, the participants emphasised, public awareness campaigns and education are needed to change these perceptions and promote the benefits of palliative care, here discussed by two different cancer leads:

Yes, I think it’s both about knowledge and, unfortunately, I think it’s also about wanting it, and this group of patients isn’t perhaps the one that shouts the loudest. They don’t shout the loudest and neither do their family members – they just don’t have the strength and energy.

[Group discussion 3, participant A]

[Participant B responds]:

I think we have to increase the general public’s awareness of palliative care and what it is. I think we have to raise public awareness about early integration being what we want to achieve. I don’t think it’s really the patients’ knowledge we need to increase – I don’t think that. And then we obviously need to improve in, for example, elderly care. I think that’s where it’s fails, although, of course, it might not be directed that much at this group of patients, but in cancer care it’s a lot to do with the public – we must increase the decision-makers’ level of knowledge.

### Challenges and complexities of providing palliative care in a fragmented healthcare system

Discussions with the participants focused on the general challenges related to palliative cancer care (i.e. both non-specialised and specialised) within a healthcare system they described as fragmented rather than merely decentralised. Participants highlighted that the involvement of multiple care providers and principals, such as regions, municipalities, and different specialists, creates a fragmented system with unclear responsibilities. This fragmentation, i.e. rapid structural changes and break down of organisations or insufficient collaboration between responsible parties complicates the coordination and delivery of palliative care, leading to inconsistent practices and gaps in palliative cancer care:

There’s a gap between municipal primary care and specialist care so the collaboration doesn’t really appear to be joined up there.

[Group discussion 2, participant A]

[Participant B responds]:

I mean, there absolutely are failures in handover communication, so I have to say it really surprises me – I didn’t think such a large proportion of cancer patients still die in hospital.

Financial constraints and resource allocation were described as constant obstacles. Financial incentives, participants stated, had led to a proliferation of health care entities, resulting in varying quality and inequalities, with some regions, or areas within regions, experiencing under-establishment and others over-establishment:

And then of course [name of region] introduced care options in specialist palliative care, and it was politically motivated – you could say it was an ideological decision. But it’s also about increasing the number of actors in this care sector and increasing the number of actors geographically. This means there are many actors working in specialised palliative care in [name of region], and a lot of them are private and then there are not-for-profit organisations… [pauses] So you could say this has generated competition to get patients. There’s been a driving force to be quick about getting on board once the referrals come and obviously this has been important in having the option to prescribe and receive advanced home care… quite simply, there’s been an incentive for care providers to be expansive and have lots of patients.

[Individual interview 1]

Changes in priorities or well-intentioned re-allocation of resources and organisational changes were often described as leading to the fragmentation of palliative care, including the dismantling of successful examples:

Yes, unfortunately I’m living in a reality here in [name of region] where they shut down specialised home care about a year ago. We might be into the second year now – I don’t quite remember – but it was recently. So they’ve completely shut down ASIH activities, which is a real shame because we’re already noticing that we’re not getting patients home – there’s a sort of half measure – these patients have to go home with ordinary home care, and then there’s a palliative resource team that’s kind of like a half measure, only available day time, and of course you can’t get that to work because they’re in so much need. Unfortunately, that’s how it is, and someone made it that way… previously it was only in [one local part of the region] so this is some kind of fairness thing.

[Group discussion 9]

Several group discussions highlighted this issue, noting that such fragmentation is due to governing bodies’ lack of collaboration with RCCs and other regional knowledge governance organisations.

Participants frequently mentioned a presumed variability in availability and quality of specialised palliative care services between regions. They noted that urban areas tend to have more resources and specialised services, while rural and sparsely populated areas face significant challenges in providing adequate palliative care:

I’m not surprised it looks like this. This pretty much reflects my view of things. Then sometimes you can think, yes, but the hospitals, the fact they have such a large share, then in the best of worlds that would be because they have institutional palliative care facilities, but that isn’t the case in many of these hospitals, for example in [name of region]. There’s no hospital up here that has institutional palliative facilities. So, those patients have been getting worse care, and not the most specialists, and this is something we know about and it’s deplorable that it is like this. And I do think it’s a bit of a failure, quite a big failure on the part of decision-makers and implementers but primarily our regional politicians, that it’s got like this. They should take far, far more responsibility.

[Individual interview 3]

This disparity was interpreted as a key explanation for the results from previous studies presented at the beginning of the discussions. Participants asserted that there are no mandatory guidelines or models for palliative care implemented, as well as an absence of defined minimum requirements for hospices, palliative care beds, homecare teams, palliative consultation teams, and digital alternatives. According to the participants, this is because each region and municipality makes priorities based on their financial situation. They also believed that the open market for services and free choice of care for patients in the capital region has significantly impacted the number of specialised palliative home care providers, and that conversely, the northern region struggles with rurality, geographical distance, and a shortage of specialised palliative care services. The participants noted that the lack of national governance and directing policies for palliative care had led to inconsistent practices and regional disparities:

There has never been any gold standard in Sweden. All six of these regions have been structured differently and managed differently. If, there’s a good example on a regional level in [name of region] then they’ve never taken that [name of another region] as exemplary; instead, they’ve organised the care according to their own management needs, that’s to say, patched it up as and when the situation dictates.

[Individual interview 2]

Several participants emphasised the importance of local initiatives and the efforts of dedicated individuals in developing specialised palliative care services in certain regions. However, they also stressed that relying solely on local efforts is not sustainable. National directive policies and coordinated efforts that can be uniformly implemented across regions were regarded as vital.

The RCCs and governmental knowledge organisations are intended to facilitate cooperation within and between regions. The study participants voiced concerns about an imbalance of power in the governance and organisation of palliative cancer care, with patients and their families underrepresented in steering groups. They said that patient representatives currently have minor impact on governance and organisation and generally stressed the need for clearer governance structures, better resource allocation, and greater inclusion of patient perspectives to address disparities and improve the quality and organisation of palliative care in cancer care.

### Policy impact and ownership problem

Participants highlighted the non-directive nature of national palliative care policy in Sweden, noting that it does not impose mandatory requirements. They identified a lack of ownership for palliative cancer care, as the responsibility for creating national cancer care guidelines does not lie with The National Board of Health and Welfare, but rather with knowledge management organisations and RCCs between which cross-regional collaborations were described as poor:

I think one thing that surprised me a bit about RCC work, and that I’ve also suggested they start changing, is that those of us working as RCC or in the different regions must work together. So, there should be a national RCC group for [specific cancer type] that follows a model of palliative care. I mean, it’s the only way, otherwise we’re sitting here doing the same things, perhaps, or different things when someone has come up with something better… We’d done some work down here to improve things for our patients locally at the different hospitals. We went round talking to them and wrote memos and we’ve shared these with the other regions that we’ve had meetings with – that way you achieve more equality – so it really surprises me that it’s not already in place and I’ve also conveyed that to our RCC heads, like – this is what has to happen. Because if there had been a national RCC group where everyone was on board with palliative care, then they would have been able to cooperate and then implement it.

[Individual interview 7]

Participants problematised that due to the absence of mandatory requirements, regions and municipalities have the autonomy to decide how much and what to invest in palliative care, allowing them to determine their own priorities and methods for organising and providing these services:

Yes, I think most of the documents [refers to policy documents for palliative care] are really, good, but one big problem, for example, the national care programme, is flexibility. We’re not allowed to write ‘shall’ and can only write ‘should’ because it isn’t a legal text and that means that our 21 regions [refers to geographical regions in Sweden] can interpret this any way they like.

[Group discussion 3]

To promote more equal opportunities for palliative care nationwide, participants suggested integrating a national minimum requirement and enhanced mandatory requirements for palliative care in national policy documents. They also recommended enhanced inclusion of palliative care in cancer type-specific guidelines, encompassing directives for early introduction of palliative care, cancer type-specific treatments and end-of-life conversations in non-specialised settings and throughout the extended care chain. Participants believed that embedding palliative care in cancer care guidelines would not only enhance the potential for making it a standard practice, but also potentially increase the knowledge level of healthcare professionals in the cancer context.

Participants suggested specifying requirements and systematically following up on targeted indicators for palliative cancer care to enable transparent comparisons between different regions. Some also recommended that policy and governance directives include strategies for systematic and continuous education of healthcare professionals about palliative cancer care and its integration in oncology across the country and in all healthcare settings where patients with potential palliative care needs are encountered.

## Discussion

This study, based on the perspectives of cancer care leads, revealed three conditional patterns that significantly shape the governance and organisation of palliative cancer care in Sweden. These patterns, highlighting multi-level knowledge gaps, the challenges of a fragmented healthcare system, and vague policy impact and ownership issues, are interdependent.

Significant challenges in palliative cancer care due to a fragmented healthcare system and non-directive national policies were highlighted. The involvement of multiple care providers and principals, such as regions, municipalities, and various specialists complicates the coordination and delivery of palliative care, leading to inconsistent practices and gaps in the ways palliative care needs are met. Financial constraints and resource allocation were consistently described as obstacles, leading to inequalities in service provision, which has also been put forward in other studies [13]. Urban areas tend to have more resources and specialised services, while rural areas face significant challenges. Further, the absence of mandatory guidelines allows regions their own interpretation of national care programs, resulting in varied adherence and implementation.

Many of the challenges disclosed regarding the state of cancer care in Sweden relate back to ownership of the problem. While the cancer care leads in this study raised the issue of professional knowledge and agency for early introduction of or referral to palliative care, their discussions most often explicitly revolved around specialised palliative care, while there was more implicit mention of non-specialised palliative care. The participants blamed politicians and governing bodies, stating there is insufficient understanding of the necessary skills, resources and responsibilities required to ensure equitable and timely access to palliative cancer care. This seems to be a major barrier to effective organisation and decision-making at all levels within the governance of the healthcare system, as well as to the provision and performance of care. The person-centred and integrated healthcare reform [36] in Sweden puts immense pressure on SALAR as a uniting force, and on independent regions and municipalities to collaborate in developing a well-structured organisation of non-specialised and specialised palliative care services that are not sensitive to shifts in political government and that have the capacity to ensure all patients with advanced cancer receive early integration of palliative care according to their needs, regardless of whether they are cared for at home, in a nursing home, or in hospital.

The consensus-based, global common sense oncology movement [41] was developed from the “grass root level” with the mission to ensure that cancer care focuses on outcomes that matter to patients. Among other things, the guiding principles highlight that quality cancer care is a fundamental human right and that care should focus on the needs of patients and society, with active involvement from patients and the public in policymaking. Further, treatments should significantly enhance survival or quality of life, and decisions should be made collaboratively between patients and healthcare professionals, integrating psychosocial oncology, survivorship, and palliative care in a timely manner. Research from randomised controlled trials and meta-analyses shows that early integration of specialised palliative care may enhance symptom relief and quality of life for patients with advanced cancer. However, the tested strategies or models typically categorised by care setting and referral method are more or less effective, and still dependent on referring individual oncologists or clinics [42], which was also voiced in this study. The participants in our study commented further that early referrals to specialised palliative care services are frequently rejected. It could be questioned; however, to what extent early integrated palliative care require specialist palliative care competency. Still, our participants stated that oncologists and other cancer care specialists (i.e. non-specialists in palliative care) are often reluctant to introduce palliative care early. This mirrors the findings of another Swedish study about attitudes and practices among Swedish physicians regarding referrals, integration, and transition between oncology care and palliative care. The majority were positive about introducing palliative care early in the illness trajectory, but in practice, few did [43]. Automatic referral, applying more-selective criteria and based on a combination of time from diagnosis, patient prognosis and personal care needs, has been suggested to streamline referral practices [44]. However, this relates to the problem of knowledge and ownership, i.e. agency for the delivery of non-specialised palliative care in the cancer context.

To promote equitable access to palliative care and avoid adequate palliative cancer care being dependent on “enthusiastic advocates,” it is crucial to regulate the governance and organisation of palliative care through more directing policy and clearer national standards for a minimum requirement of resource allocation for palliative care. In December 2025, new national guidelines for palliative care were presented, providing clearer direction for key health care stakeholders [45]. Although the guidelines represented an improvement and outlined prioritised areas, they remained advisory in nature and lacked explicit guidance on implementation and follow up. One of the prioritised areas in the document was to organise systematic training in non-specialised palliative cancer care (including training in difficult communication skills) for healthcare professionals in all settings, i.e. hospital cancer care settings, primary care and nursing homes. We argue for national-level collaborative efforts to develop this training, instead of relying on regions or municipalities to independently create parallel initiatives.

Public and political awareness campaigns are crucial for promoting the benefits of palliative care and informing about national policies, as well as dissipating the stigma associated with palliative care, i.e. that it solely comprises end-of-life care. Political awareness campaigns can influence policymakers to prioritise palliative care and allocate reasonable funding, resources, and integration of palliative care services within the healthcare system. Finally, public campaigns can foster community support and involvement in palliative care initiatives [28]. Further, establishing clear standards for the organisation and allocation of resources enabling the integration of palliative care early in the illness trajectory of people with advanced cancer is essential. This involves drawing up guidelines to ensure that palliative care services are well-structured and adequately funded. It also includes directives for national collaborative spaces for regions and municipalities and their knowledge management organisations, cancer and palliative care leads, patient representatives, and governance bodies. One concrete example put forward by our study participants is a national RCC-collaboration structure. These standards should be part of a strengthened policy directive that provides a framework for politicians, regional authorities, and municipal authorities to follow.

However, even with a strengthened policy directive, is challenging in complex systems. Implementation research cautions that guidelines and pathways rarely generate consistent practice change on their own. Adoption and sustained use depend on multi-level contextual factors (e.g., resources, leadership, professional norms, and inter-organisational coordination) and typically require context-sensitive implementation strategies, including implementation support, accountability, and iterative evaluation [46–48]. Hence, directives for the use of RE-AIM, Medical Research Council evaluation of complex interventions, and WHO constructs of health service evaluation frameworks are suggested [49].

### Study limitations

Willingness to participate in this study was low (23%), with time constraints given as the primary reason for non-participation. However, the thirty-six participants in this study do represent the desired diversity in terms of region, profession, assignment, and cancer type. The idea behind presenting the results from our previous studies to trigger interviews and group discussions was to initiate conversations about governance and organisation of palliative cancer care based on current knowledge about the subject. Although place of death as an indicator of governance and organisation has its weaknesses, when considered in relation to current Swedish health care reforms, legislation, and the palliative care discourse, including population preferences, the framing of results about place of death patterns triggered reflective group discussions about the national palliative care governance.

Had the participants been provided with this information prior to their scheduled interviews, it is possible that they would have been better prepared and potentially may have reflected more and provided fuller insights.

## Conclusion and implications

This study has revealed significant challenges in national palliative cancer care governance, primarily due to a multilevel knowledge gap about palliative care, a fragmented healthcare system and non-directive national policies. Addressing these challenges requires integration of mandatory national minimum requirements for palliative care in national policy, and clearer standards for palliative care resource allocation. Furthermore, comprehensive strategies and coordinated efforts that can be uniformly implemented across regions, including systematic training for healthcare professionals, public and political awareness campaigns, and establishing national collaborative spaces for regional cancer care leads are essential to ensure equitable and timely access to palliative care for all patients with advanced cancer.

## Electronic Supplementary Material

Below is the link to the electronic supplementary material.

Supplementary Material 1

## Data Availability

We acknowledge the use of data and materials to be freely available under the Creative Commons Attribution (CC BY) licence, which allows for unrestricted use, distribution and reproduction, provided the original authors are credited. The datasets used analysed during the current study are available from the corresponding author on reasonable request.

## Abbreviations

WHO: World Health Organisation
NBHW: National board of health and welfare
SALAR: Swedish association of local authorities and regions
RCC: Regional cancer centre

## References

[1] World Health Organisation. Global patient safety action plan 2021–2030: towards eliminating avoidable harm in health care. World Health Organisation. 2021. https://www.who.int/publications/i/item/9789240032705. Accessed 31 Mar 2025.

[2] Bray F, Laversanne M, Sung H, Ferlay J, Siegel RL, Soerjomataram I, et al. Global cancer statistics 2022: GLOBOCAN estimates of incidence and mortality worldwide for 36 cancers in 185 countries. Cancer J Clin. 2024;74:229–63. 10.3322/caac.21834 38572751

[3] Bizuayehu HM, Ahmed KY, Kibret GD, Dadi AF, Belachew SA, Bagade T, Tegegne TK, Venchiarutti RL, Kibret KT, Hailegebireal AH, Assefa Y. Global disparities of cancer and its projected burden in 2050. JAMA Netw open. 2024;7:e2443198. 39499513 10.1001/jamanetworkopen.2024.43198 PMC11539015

[4] World Health Organisation. WHO report on cancer: setting priorities, investing wisely and providing care for all. World Health Organisation. 2020. https://www.who.int/publications/i/item/9789240000000. Accessed Mar 31 2025.

[5] Ferrell BR, Temel JS, Temin S, Alesi ER, Balboni TA, Basch EM, et al. Integration of palliative care into standard oncology care: American Society of Clinical Oncology clinical practice guideline update. J Clin Oncol. 2017;35:96–112. 28034065 10.1200/JCO.2016.70.1474

[6] Nadolny S, Schildmann E, Gaßmann ES, Schildmann J. What is an early palliative care intervention? A scoping review of controlled studies in oncology. Cancer Med. 2023;12:21335–53. 37902232 10.1002/cam4.6490 PMC10726823

[7] Jordan K, Aapro M, Kaasa S, Ripamonti C, Scotté F, Strasser F, et al. European Society for Medical Oncology (ESMO) position paper on supportive and palliative care. Ann Oncol. 2018;29:36–43. 29253069 10.1093/annonc/mdx757

[8] Huo B, Song Y, Chang L, Tan B. Effects of early palliative care on patients with incurable cancer: A meta-analysis and systematic review. Eur J Cancer. 2022;31:e13620. 10.1111/ecc.13620 35612356

[9] Strang P. Palliative oncology and palliative care. Mol Oncol. 2022;16:3399–409. 35762045 10.1002/1878-0261.13278 PMC9533690

[10] Swenne JIE, Hansen TF, Nissen RD, Steffensen KD, Stie M, Søndergaard J, et al. Early integration of basic palliative care in cancer: scoping review of cross-sectorial models–components, facilitators, barriers. BMJ Support Palliat Care. 2024;14:e2349–65. 38663981 10.1136/spcare-2023-004651

[11] Han PK, Rayson D. The coordination of primary and oncology specialty care at the end of life. JNCI Monogr. 2010;2010:31–7. 10.1093/jncimonographs/lgq003 PMC3482948 20386052

[12] Kaasa S, Loge JH, Aapro M, Albreht T, Anderson R, Bruera E, et al. Integration of oncology and palliative care: a Lancet Oncology Commission. Lancet Oncol. 2018;19:e588–653. 30344075 10.1016/S1470-2045(18)30415-7

[13] Sørensen DM, Dalton SO, Egholm CL, Bidstrup P, Brodersen JB, Rosted E. Barriers and facilitators to national guideline implementation for palliative cancer care in a Danish cross-sectoral healthcare setting: A qualitative study of healthcare professionals’ experiences. Psycho‐Oncology. 2024;33:e6267. 38078707 10.1002/pon.6267

[14] Sánchez-Cárdenas MA, Garralda E, Arias-Casais NS, Sastoque ERB, Van Steijn D, Moine S, et al. Palliative care integration indicators: an European regional analysis. BMJ Supp Palliat Care. 2024;14:e1041–8. 10.1136/bmjspcare-2021-003181 34518283

[15] National Board of Health and Welfare. National guidance for good palliative care. Stockholm. 2012. https://www.socialstyrelsen.se/globalassets/sharepoint-dokument/artikelkatalog/nationella-riktlinjer/2012-5-1.pdf. Accessed Mar 31; 2025.

[16] Regional Cancer Centres in collaboration. National guidelines for palliative care. Sweden: Regional Cancer Centres in collaboration. National care program for palliative care [in Swedish]. Sweden2021.Accessed Mar 31; 2025.

[17] O’Sullivan A, Carling L, Öhlén J, Nyblom S, Ozanne A, Hedman R, et al. Palliative care in policy documents for adults with cancer and non-cancer diseases with potential palliative care needs: a document analysis. Palliat Care Soc Pract. 2024;18:26323524241296145. 39634193 10.1177/26323524241296145 PMC11615978

[18] Cohen J, Deliens L, editors. A public health perspective on end of life care. Oxford: Oxford University Press; 2012.

[19] Hoare S, Antunes B, Kelly MP, Barclay S. End-of-life care quality measures: beyond place of death. BMJ Support Palliat Care. 2022;14:613–21. 10.1136/spcare-2022-003841 35859151

[20] Fereidouni A, Rassouli M, Salesi M, Ashrafizadeh H, et al. Preferred place of death in adult cancer patients: a systematic review and meta-analysis. Front Psychol. 2021;12:704590. 34512460 10.3389/fpsyg.2021.704590 PMC8429937

[21] Cabañero-Martínez MJ, Nolasco A, Melchor I, Fernández-Alcántara M, et al. Place of death and associated factors: a population-based study using death certificate data. Eur J Public Health. 2019;29:608–15. 30601984 10.1093/eurpub/cky267

[22] Öhlén J, Stina N, Anneli O, Stefan N, Hanna G, Johan FC, et al. Influence of palliative care policy on place of death for people with different cancer types: a nationwide’register study. PLoS ONE. 2025;20:e0320086. 40146710 10.1371/journal.pone.0320086 PMC11949374

[23] Larsdotter C, Nyblom S, Gyllensten H, Furst CJ, Ozanne A, Hedman R, et al. Trends in the place of death in Sweden from 2013 to 2019 - disclosing prerequisites for palliative care. Palliat Care Soc Pract. 2024;18:26323524241238232. 38497045 10.1177/26323524241238232 PMC10943753

[24] Cohen J, Pivodic L, Miccinesi G, Onwuteaka-Philipsen B, Naylor W, Wilson D, et al. International study of the place of death of people with cancer: a population-level comparison of 14 countries across 4 continents using death certificate data. Br J Cancer. 2015;113:1397–404. 26325102 10.1038/bjc.2015.312 PMC4815784

[25] Sarmento VP, Higginson IJ, Ferreira PL, Gomes B. Past trends and projections of hospital deaths to inform the integration of palliative care in one of the most ageing countries in the world. Palliat Med. 2016;30:363–73. 26163531 10.1177/0269216315594974 PMC4800459

[26] Bajwah S, Oluyase AO, Yi D, Gao W, Evans CJ, Grande G et al. The effectiveness and cost-effectiveness of hospital‐based specialist palliative care for adults with advanced illness and their caregivers. Cochrane database Syst Rev. 2020. 10.1002/14651858.CD012780.pub2 PMC8428758 32996586

[27] Szilcz M, Wastesson JW, Morin L, Calderón-Larrañaga A, Lambe M, Johnell K. Potential overtreatment in end-of-life care in adults 65 years or older dying from cancer: applying quality indicators on nationwide registries. Acta Oncol. 2022;61:1437–45. 36495144 10.1080/0284186X.2022.2153621

[28] National Board of Health and Welfare. Statistik om dödsorsaker 2019 (Causes of death statistics 2019). Sweden. 2020. https://www.socialstyrelsen.se/globalassets/sharepoint-dokument/artikelkatalog/statistik/2020-6-6798.pdf. Accessed Mar 31 2025.

[29] Abel J, Kellehear A, Karapliagou A. Palliative care-the new essentials. 2018. 10.21037/apm.2018.03.04 29764169

[30] Centeno C, Garralda E, Carrasco JM, den Herder-van M, Aldridge M, Stevenson D, et al. The palliative care challenge: analysis of barriers and opportunities to integrate palliative care in Europe in the view of national associations. J Palliat Med. 2017;20:1195–204. 28509657 10.1089/jpm.2017.0039

[31] den Herder-van, der Eerden M, Ewert B, Hodiamont F, Hesse M, Hasselaar J, Radbruch L. Towards accessible integrated palliative care: perspectives of leaders from seven European countries on facilitators, barriers and recommendations for improvement. J Integr Care. 2017;25:222–32. 10.1108/JICA-03-2017-0006 PMC5868545 29720896

[32] Woitha K, Carrasco JM, Clark D, Lynch T, Garralda E, Martin-Moreno JM, et al. Policy on palliative care in the WHO European region: an overview of progress since the Council of Europe’s (2003) recommendation 24. Eur J Public Health. 2016;26:230–5. 26545804 10.1093/eurpub/ckv201

[33] Clark D, Baur N, Clelland D, Garralda E, López-Fidalgo J, Connor S, et al. Mapping levels of palliative care development in 198 countries: the situation in 2017. J Pain Symptom Manag. 2020;59:794–807. e4. 10.1016/j.jpainsymman.2019.11.009 PMC7105817 31760142

[34] Arias-Casais N, Garralda E, Rhee J. EAPC atlas of palliative care in Europe. Romania. 2019;122.

[35] Thorne S. Interpretive description. Qualitative research for applied practice. 2 ed. New York & London: Routledge, Taylor & Francis group; 2016.

[36] Sacchetti L, Lindahl G. Understanding Healthcare Design Transformations. Insights from the Swedish Experience. Stud Health Technol Inf. 2024;319:237–491879. 10.3233/SHTI240947 39618364

[37] Regional Cancer Centres in Sweden. National guidelines for palliative care. 2026. https://cancercentrum.se/samverkan/regional-cancer-centres. Accessed Mar 31 2025. Accessed April 21 2026.

[38] Öhlén J, Nyblom S, Ozanne A, Hedman R et al. Förutsättningar för jämlik personcentrerad palliativ vård. (Prerequisites for equal person-centred palliative care). 2025.

[39] Swedish Regional Cancer Centres in Collaboration. Personcentrerat och sammanhållet vårdförlopp (National person-centred and coordinated care pathway for palliative care). 2022. https://cancercentrum.se/samverkan/regional-cancer-centres. Accessed Mar 31 2025.

[40] Martinsson L, Heedman PA, Lundström S, Axelsson B. Improved data validity in the Swedish Register of Palliative Care. PLoS ONE. 2017;12:e0186804. 29049396 10.1371/journal.pone.0186804 PMC5648220

[41] Booth CM, Sengar M, Goodman A, Wilson B, Aggarwal A, Berry S, et al. Common Sense Oncology: outcomes that matter. Lancet Oncol. 2023;24:833–5. 37467768 10.1016/S1470-2045(23)00319-4

[42] Aldridge MD, Hasselaar J, Garralda E, van der Eerden M, Stevenson D, McKendrick K, et al. Education, implementation, and policy barriers to greater integration of palliative care: A literature review. Palliat Med. 2015;30:224–39. 26405109 10.1177/0269216315606645

[43] Adolfsson K, Kreicbergs U, Bratthäll C, Holmberg E, Björk-Eriksson T, Stenmarker M. Referral of patients with cancer to palliative care: Attitudes, practices and work‐related experiences among Swedish physicians. Eur J Cancer. 2022;31:e13680. 10.1111/ecc.13680 PMC10909424 35965390

[44] Bruera E, Hui D. Integrating supportive and palliative care in the trajectory of cancer: establishing goals and models of care. J Clin Oncol. 2010;1:28. 10.1200/JCO.2010.29.5618 20660825

[45] National Board of Health and Welfare. National guidelines for palliative care. Stockholm: National Board of Health and Welfare; 2025.

[46] Grol R, Grimshaw J. From best evidence to best practice: effective implementation of change in patients’ care. Lancet. 2003;11:1225–30. 10.1016/S0140-6736(03)14546-1 14568747

[47] Wang T, Tan JB, Liu XL, Zhao I. Barriers and enablers to implementing clinical practice guidelines in primary care: an overview of systematic reviews. BMJ Open. 2023;13(1):e062158. 36609329 10.1136/bmjopen-2022-062158 PMC9827241

[48] Büscher A, Kugler J. The effectiveness of clinical pathways in inpatient settings-an umbrella review. J Public Health. 2025;33:2717–30.

[49] Rizvi F, Wilding HE, Rankin NM, Le Gautier R, Gurren L, Sundararajan V, et al. An evidence-base for the implementation of hospital-based palliative care programs in routine cancer practice: A systematic review. Palliat Med. 2023;37:1326–44. 37421156 10.1177/02692163231186177 PMC10548767

[50] Association WM. World Medical Association Declaration of Helsinki: ethical principles for medical research involving human participants. JAMA. 2025;333:71–4. 39425955 10.1001/jama.2024.21972

