# Supplementary material for: Regional cancer care leads´ and patient representatives´ perspectives on national governance and organisation of palliative cancer care
Source: BMC Health Serv Res. 2026 May 13;26:699. doi: 10.1186/s12913-026-14691-z (PMC13173704; doi:10.1186/s12913-026-14691-z)
Supplement: Supplementary file 1 — Supplementary Material 1 [file 12913_2026_14691_MOESM1_ESM.docx]

**National governance and organisation of palliative cancer care**

Thematic questions:

- How do you view these results that we have presented, from our two previous studies aimed at: 1) investigate trends in place of death for people dying of cancer between 2013-2019; and 2) explore the inclusion of palliative care relevant information in cancer type specific national guidelines, based on the national healthcare goal of equal care?
- What can affect the conditions for palliative cancer care nationally, and potentially explain regional differences in place of death?
- What impact does the national knowledge governance of health care for palliative care have?
- What strategies do you see as successful for governance of palliative care within the region and cancer type you represent?

Probing questions and follow-up individual interviews intend to clarify or further elaborate participants’ statements and topics being discussed during group discussions and first-time single interviews.
